# Causes and Recommendations for Fever in Sickle Cell Pediatric Patients in the Emergency Department: A Single-Center Study

**DOI:** 10.7759/cureus.44959

**Published:** 2023-09-09

**Authors:** Manal A Halwani

**Affiliations:** 1 Pediatric Emergency, King Abdulaziz University Hospital, Jeddah, SAU

**Keywords:** length of hospitalization, bacterial infections, pediatric emergency department (ped), periodic fever, pediatric sickle cell disease

## Abstract

Background

Children with sickle cell disease (SCD) are prone to bacterial infections, culminating in life-threatening incidences. Early evaluation of children with SCD helps in effective management and support.

Methodology

A retrospective study was conducted using medical records for febrile episodes in SCD children ≤14 years of age who presented to the Emergency Department of King Abdulaziz University Hospital, Jeddah, Kingdom of Saudi Arabia from 2015 to 2018. A total of 304 episodes were encountered in the Emergency Department during this period.

Results

The clinical diagnosis included confirmed bacterial infection (4.5%), presumed bacterial infection (24.6%), and those without bacterial infection (57.5%). The incidence of bacteremia was found in 3.0% of the episodes and urinary tract infection in 1.5%. The most common isolates were *Staphylococcus aureus*, *Streptococcus viridians*, *Salmonella *species, and *Escherichia coli*. Overall, 52% of the febrile episodes resulted in hospitalization, of which 74% had at least one prior hospitalization. The hospitalization probability across the two sexes was statistically insignificant (p = 0.029). The likelihood of admission increased with age (p < 0.001) and temperature (p < 0.001). The study included 140 children with SCD who had at least one abdominal sonogram performed at our hospital between 2015 and 2018. There were changes in the radiographic appearance of the spleen in patients with SCD who were expected to undergo autosplenectomy between the ages of five and 17 years.

Conclusions

The study envisages the risk associated with febrile episodes and the prompt recovery of such patients through clinical confirmations. Parents should be aware and observant of the complications of infectious illnesses for speedy medical assistance.

## Introduction

Sickle cell disease (SCD) is an inherited autosomal recessive disorder that affects hemoglobin [1]. A genetic hematological disorder, SCD affects 7% of the global population and 4.2% of Saudi Arabia’s population [2,3]. Fever is a significant condition in children with SCD, often indicating serious underlying conditions requiring medical attention [4]. SCD children face an increased risk of invasive infection, morbidity, and mortality due to splenic dysfunction [5]. Children with severe chronic pulmonary disease are more susceptible to fatal infections due to dysfunctional antibodies and febrile conditions. Despite penicillin prophylaxis and pneumococcal conjugate vaccines reducing gram-positive pneumococcus infections, antibiotic-resistant genes and bacterial strains have increased the risk of infection, making them more vulnerable [6,7].

SCD patients are 10-100% more susceptible to invasive pneumococcal disease, with fatality rates of 15% [8,9]. Bacterial sepsis caused by encapsulated bacteria remains a significant concern. Patients presenting with fever ≥38.5°C should receive broad-spectrum antibiotics within 60 minutes of triage [10]. Patients with SCD and high temperatures require hospitalization for antibiotic therapy [11]. Children with SCD are at higher risk of urinary tract infections, particularly severe bacterial infections due to reduced spleen function. Repeated sickling and infarction in splenic patients with SCD often require autosplenectomy by the age of 5-17 years. SCD patients are susceptible to encapsulated bacteria, including *Streptococcus pneumoniae*, *Haemophilus influenzae*, *Neisseria meningitidis*, and *Salmonella *spps. Fever in SCD patients should be treated as it may signify a dangerous bacterial infection. All fevers should be treated empirically until bacterial infection is ruled out [12].

Splenic sequestration is a potentially fatal syndrome caused by SCD, affecting children and causing spleen growth and reduced oxygen-carrying red blood cell (RBC) levels. In early childhood, enlarged spleen atrophies due to vascular occlusion, infarction, and auto-splenectomy, resulting in severe complications [13]. A shrunken spleen is defined as having a long axis of less than 50 mm, but splenomegaly can persist into adulthood. Autosplenectomy is a secondary outcome in SCD patients using abdominal ultrasonography [14]. SCD patients often require hospitalization for intravenous antibiotic therapy, but these visits can be costly for families. For low-risk sepsis patients, outpatient therapy is recommended for effective management. Outpatient follow-up with pediatric hematologists is associated with better patient outcomes and reduced rehospitalization rates [15].

This study investigates fever causes and outcomes in children with SCD in the Emergency Department (ED) at King Abdulaziz University Hospital (KAUH), guiding clinicians and seeking a modifying clinical pathway for the current situation.

## Materials and methods

Study population

A retrospective study was conducted using the medical records of the ED at KAUH, Jeddah for SCD pediatric patients aged ≤14 years from 2015 to 2018. This study was approved by the Unit of Biomedical Ethics of Research Committee, Unit of Medicine, King Abdulaziz University, Kingdom of Saudi Arabia (reference number: 603-18). A review of the patient’s medical records was performed for a total of 304 patients. The study aimed to evaluate the causes and clinical outcomes of SCD children who presented to the ED and to explore improved clinical pathways that can be used in KAUH.

Data collection

The patient’s complaints were extracted from the records. All patients ≤14 years old and with documented fever ≥38.5°C were included. The patients’ modes of arrival, episodes of fever, previous clinical history, and diagnostic testing including a complete blood count, blood culture, urine analysis, and sputum culture were recorded. The primary care processes of interest were the receipt of specific diagnostic testing, treatment, and disposition. Finally, hospital disposition was classified as admitted or discharged from the ED. The physician determined hospital admission and recommended for patients with a fever above 38.5°C. Admitted children were treated (parenteral or oral) with ceftriaxone (100 mg/kg/day for seven days) or until a negative culture was obtained.

Investigation of clinical events

SCD-related complications were evaluated based on clinical and laboratory records. Fever presentations were categorized as the same episodes at an interval of 7-10 days. A confirmed bacterial infection was defined as a positive blood culture with the identified organism. Episodes clinically suspected to be bacterial infections but without any identified organism were categorized as presumed bacterial infections. Episodes confirmed as non-bacterial infections were attributed to viral infections or atypical organisms. The repeated episodes of sickling and infarction led to autosplenectomy between the ages of five and 17.

Statistical analysis

The data were entered and encoded using SPSS statistical software version 25 (IBM Corp, Armonk, NY, USA). All quantitative variables are presented by mean + SD and qualitative variables as frequency and percentages. The association between demographic variables, presenting complaints, blood transfusion, and fever status (yes/no) was analyzed by the chi-square test. The mean difference in hematological findings among both groups was compared using the independent-sample t-test. P-values <0.05 were considered significant.

## Results

Patients/episodes

A total of 304 patients presented to the ED. Of these, 118 (38.8%) patients with fever were included in the study. Among the 118 (38.8%) patients with fever, there were 52 (44.1%) males and 66 (55.9%) females. The mean age of patients with fever was 7.84 ± 3.6 and those without fever was 9.68 ± 3.41. The maximum number of patients were from Saudi Arabia (189). The most encountered complaints were body aches and severe pain (fever = 63 (53.4), without fever = 123 (66.1%). One to three emergency room visits were most common (fever = 114 (96.6), without fever = 183 (98.4%). Demographic data of patients who presented during primary diagnosis with fever are shown in Table 1.

**Table 1:** Characteristics of children with sickle cell disease presenting to the Emergency Department with febrile episodes. Data are presented by frequency (%), and a p-value <0.05 is considered significant. SCD: sickle cell disease; ER: emergency room

Demographics	SCD f (%)		
	With fever, n = 118 f (%)	Without fever, n = 186 f (%)	P-value
Gender			
Male	52 (44.1)	87 (46.8)	0.644
Female	66 (55.9)	99 (53.2)	
Nationality			
Saudi	75 (63.6)	114 (61.3)	0.691
Non-Saudi	43 (36.4)	72 (38.7)	
Age (years)	7.84 ± 3.6	9.68 ± 3.41	˂0.001
Presenting complaints			
Headache	1 (0.8)	8 (4.3)	0.029
Chest pain	13 (11.0)	14 (7.5)	
Back pain, bone ache, body ache, hip pain, hand and foot, other pain	63 (53.4)	123 (66.1)	
Abdominal pain	7 (5.9)	5 (2.7)	
Pallor	16 (13.6)	23 (12.4)	
Shortness of breath	14 (11.9)	10 (5.4)	
Seizure	2 (1.7)	0 (0.0)	
Palpitation	0 (0.0)	2 (1.7)	
Jaundice	2 (1.7)	1 (0.5)	
Number of ER visits			
1–3 visits	114 (96.6)	183 (98.4)	0.314
4–6 visits	4 (3.4%)	3 (1.6)	
Previous blood transfusion			
No transfusion			0.70
1–3	21 (17.8)	51 (27.4)	
<5	90 (76.3)	131 (70.4)	
>5	4 (3.4)	1 (0.5)	

Diagnoses

The clinical diagnoses included confirmed bacterial infection (3.0%), presumed bacterial infection (24.6%), and without bacterial infection due to viral or unknown etiologies (72.4%) (Table 2).

**Table 2:** Distribution of the clinical diagnosis of patients. Data are presented by frequency (%).

Clinical group	Diagnosis	Number of episodes
Confirmed bacterial infection (n = 6)	Bacteremia	4
	Urinary tract infection	2
Presumed bacterial infection (n = 33)	Acute tonsillitis	13
	Otitis media	12
	Bacterial conjunctivitis	8
Presumed viral infection (without bacterial infection) (n = 95)	Culture-negative fever	60
	Upper respiratory tract infection	35
Total episodes diagnosed		134

Fever

Assessment of the febrile episodes was based on primary screening, radiographic examinations, and bacteriologic cultures. Of the 118 febrile episodes, confirmed bacterial infection occurred in six (4.5%) episodes. Overall, 3% of the febrile episodes were diagnosed with bacteremia. Blood cultures were positive for *Staphylococcus aureus*, *Streptococcus viridans* infection in two children (one each), and *Salmonella *spp. in two children. None of the blood samples were positive for *Streptococcus pneumoniae*. The clinical diagnosis of fever was for viral or atypical organisms, some without any known etiology. Nearly 44.8% of the episodes have no clear explanation for the fever. Although blood and sputum cultures were negative, infection from an atypical organism is suspected (Table 3).

**Table 3:** Episodes with confirmed bacterial infections. The normal body temperature is 37.4°C (98.6°F). The reference hemoglobin level in 1-14-year-olds is 9.5-14 g/dL. The normal range of white blood cell count in those aged five months to 12 years is 5.0–12.0 x 10^9^/L. WBC: white blood cell; LOS: length of stay

Episodes	Age (years)/Sex	Temperature (℃)	Hemoglobin (g/dL)	WBC (×10^9^/L)	Organism	Discharge diagnosis, LOS
1	5 M	37.6	7.3	22.4	*Staphylococcus aureus*	Bacteremia, 15 days
2	9 M	38.0	7.1	49.5	*Streptococcus viridans*	Bacteremia, 8 days
3	7 F	37.5	6.1	13.4	*Salmonella *spp.	Bacteremia, 7 days
4	8 F	38.0	6.3	13.8	*Salmonella *spp.	Bacteremia, 7 days
5	14 F	38.0	8.5	17.6	*Escherichia coli*	Urinary tract infection, 5 days
6	10 F	37.4	7.3	21.2	*Escherichia coli*	Urinary tract infection, 7 days

Hematological findings

The study results indicated that there were no significant statistical differences in parameters such as white blood cell (WBC) count, RBC count, hemoglobin levels, reticulocyte counts, platelet counts, and hematocrit levels between the groups (p > 0.05) (Table 4).

**Table 4:** Hematological findings. Data are presented by mean ± SD, and a p-value <0.05 is considered significant. WBC: white blood cell; RBC: red blood cell; HCT: hematocrit

Variables	Fever status	Mean	SD	P-value
WBC	Fever	16.87	8.39	0.189
	No fever	15.56	7.83	
RBC	Fever	2.88	0.80	0.339
	No fever	3.07	1.98	
Hemoglobin	Fever	7.93	1.87	0.238
	No fever	7.69	1.51	
Reticulocyte count	Fever	17.83	79.74	0.318
	No fever	11.075	7.27	
Platelet count	Fever	346.9	151.3	0.283
	No fever	368.0	163.47	
HCT	Fever	22.84	5.48	0.423
	No fever	26.07	42.10	

Prevalence of medication

Medications were administered to patients based on their condition. Among the medications, penicillin was the most frequently prescribed. Specifically, it was administered to 98 patients with fever and 154 patients without fever. Another commonly used medication was paracetamol, which was given to 77 patients with fever and 101 patients without fever. In contrast, medications such as ibuprofen and morphine were predominantly used for patients without fever (Table 5).

**Table 5:** Medication for sickle cell disease in the Emergency Department.

Medication received	With fever	Without fever
Pencil/Ospen	98	154
Paracetamol	77	101
Ibuprofen	42	70
Morphine	18	44

## Discussion

The complications associated with SCD reduce the quality of life causing high morbidity and mortality. Bacterial infections have been stated as a major risk to SCD patients, manifested by high fever and lethargy. Timely assessment and management of fever is the prerequisite for caring for SCD patients and preventing lethal infections [16,17]. All patients presenting to the ED with fever were clinically diagnosed and blood cultures were obtained per standard protocol to administer broad-spectrum antibiotics after obtaining blood cultures. In this study, the common clinical diagnosis was presumed bacterial infection (24.6%).

Septicaemia and meningitis as major causes of death in SCD individuals, with over 30% attributed to encapsulated bacteria [18,19]. The study found bacteremia incidence at 3.0%, slightly higher than previous rates, and comparable to a study conducted among pediatric patients [20]. The predominant pathogens noted were *Staphylococcus aureus*, *Streptococcus viridians*, *Salmonella *spp., and *Escherichia coli*. There was no pneumococcal infection, likely due to routine PCV vaccination [21].

In this study, the rate of urinary tract infection was only 1.5%, which is far lower than previously reported rates [22]. Urine evaluation in SCD patients is crucial for diagnosing disease complications, and confirmed bacteremia patients with fever should undergo antibiotic treatment until negative blood cultures are obtained. SCD patients are vulnerable to infection because of variables such as splenic dysfunction, poor adaptive immunity, and immunological deficits caused by malnutrition. Lifelong impairments in innate, humoral, and cellular immune function result from early splenic dysfunction and starvation [23]. Parenteral antibiotics are a prerequisite for the management of SCD children with fever to prevent further complications such as meningitis. High body temperature and WBC counts are some of the indicators of bacterial infections. Previous studies have revealed that children with SCD have a 3-5% higher risk of bacterial infections due to weak immune activity [1]. The incidence of bacteremia in this study is without any associated morbidity or mortality among the affected children. WBC count has often been used as a predictor of serious bacterial infection, as well as to differentiate between bacterial and viral infections [24,25].

The study found that 52% of fever episodes required hospital admission, with 70% having a prior admission. Most patients were discharged home, with 48% following a scheduled follow-up. Outpatient therapy with oral antibiotics could be effective for uncomplicated SCD, especially for low-risk patients. Prompt management of complications and a satisfactory discharge plan can reduce hospital admission and readmission rates. Supportive care with advanced drug therapies may enhance SCD survival rates.

SCD is a global health concern with high morbidity and mortality rates. Management priorities vary by geographic and socioeconomic contexts. Resource-rich nations prioritize perinatal screening, antibiotic prophylaxis, and vaccination programs to reduce infection mortality and improve quality of life. Resource-poor environments prioritize basic care, antibiotic therapy, and transfusion therapy.

The only limitation of the study is the modest number of cases as we only evaluated patients who attended the ED or were hospitalized at KAUH. Children who presented at outpatient clinics were not included in the study.

## Conclusions

Recent studies have revealed an increasing age of children with bacteremia due to non-vaccine serotypes, discontinuation of penicillin prophylaxis, and central venous access devices, leading to higher admission rates for fever management. Patients with positive blood cultures exhibiting signs of severe illness should be treated with intravenous antibiotic therapy or as recommended by the investigator at the time of presentation. In our experience, we recommend an initial dose of intravenous ceftriaxone followed by outpatient therapy with an oral antibiotic in patients at low risk for sepsis. This may be a viable clinical pathway modification option to use in the current setting. If patients adhere to it, this clinical pathway may be cost-effective and reduce hospitalization or hospital stays or visits. The study envisages the risk associated with febrile episodes and the prompt recovery of such patients through clinical confirmations. Parents should be aware and observant of the complications of infectious illness for speedy medical assistance.
